# Supplementary material for: Patterns of Intron Gain and Loss in Fungi
Source: PLoS Biol. 2004 Nov 30;2(12):e422. doi: 10.1371/journal.pbio.0020422 (PMC532390; doi:10.1371/journal.pbio.0020422)
Supplement: Table S1 — Also available at http://genes.mit.edu/NielsenEtAl/. (4.3 MB ZIP). [file pbio.0020422.st001.zip › NielsenEtAl/html/248.html]

AN5166.1.NCU09643.1.MG09371.1.FG04237.1


```
 CLUSTAL W (1.82) Multiple Sequence Alignments - Introns Inserted


Sequence 1: MG09371.1	458 aa
Sequence 2: FG04237.1	455 aa
Sequence 3: NCU09643.1	449 aa
Sequence 4: AN5166.1	446 aa
Alignment Length: 463 aa
Number Identitical Residues: 229 aa
Alignment Score (without introns) 11686


MG09371.1 	-MTKPRRGVSFPHRKNGYSDGRRSSFSEASEDGSPTKMRSTST--LNNIQE0APPT~PQA
NCU09643.1	MTGRSRRGVKFPHS------GRRSSMSEASEESSPSKTRALAN-----IQE0KSKD~AGD
FG04237.1 	-MARSKRGVRFPNR----SDGRRSSFSSE-DGGSPTKLKSQSSGQLETVSE0KQPP~AVG
AN5166.1  	-MSKSRRSVRFQHR----SDGRRPSISDASDAASDPGSPSKNG-VVAQPAT~IPEE0KTE
          	   :.:*.* * :     :.***.*:*. :: .* .   :                    

MG09371.1 	-QAVADYEKKKANFITRTLWTFVMIAIFFGALFMGHIYIILIITAIQIISFKEVIAIANV
NCU09643.1	NQQLSDYEKKKANFIERTLWTFVMIAGFFAALFMGHIYIVVIITAVQIISFKEVIAISSG
FG04237.1 	-EKQAEYEKKKANFITRTFWTFVMFALFFTALFMGHIYIITIITAIQIVSFKEVIAIANV
AN5166.1  	APPLDEYQKKKQTFITRTIWTFVMIAGFFIAMFSGHIYIIGLVTAVQIISFKEVIAIANV
          	     :*:*** .** **:*****:* ** *:* *****: ::**:**:********:. 

MG09371.1 	PSKARSLSSAKSLNWYWLASTMYFLYGESVIYYFKHIVLVDKVLLPLATHHRFISFILYV
NCU09643.1	ASRARDIQSTKSLNWYWLAVAMYFLHGETVIYYFKHIVMVDRVLLPLATHHRFICFTLYV
FG04237.1 	PSRARSLRSTKSLNWYWLATTMYFLYGETVIYYFKHIILVDKVLLPLATHHRFISFILYV
AN5166.1  	PSKEKNIRFTKSLNWYFLGTSMYFLYGESVIYYFKHILLVDRVLLPLASHHRFISFVLWI
          	.*: :.:  :******:*. :****:**:********::**:******:*****.* *::

MG09371.1 	FG~FVFFVMSLQAGHYKFQFTNFAWTHMALYLIVVQAHFVMNNIFEGMIWFFLPAALVIT
NCU09643.1	VG1FVFFVSTLKGGHLKFQFTQFVWAHMALFLIVGQSHFIMNNVFDGLIWFFLPAALVIT
FG04237.1 	FG1FVFFVTSLQAGHYKFQFTNFAWTHMALYLIVVQAHFIMNNVFEGMIWFFLPAALVIT
AN5166.1  	MG1FVFFVASLQKGHYRFQFTQFAWTHMALYLIVVQAHFVMNNILEGMIWFFLPASLVIT
          	.* ***** :*: ** :****:*.*:****:*** *:**:***:::*:*******:****

MG09371.1 	NDIWAYICGITFGRTQLIKLSPKKTVEGFVGAWVMTVLFGILLTHFLTRSKYFICPVN0D
NCU09643.1	NDIFAYVCGITFGRTQLIKLSPKKTVEGFLGAWVMTVLFGILLTHILMRSRFFICPAN~D
FG04237.1 	NDIFAYICGIMFGRTQLIKLSPKKTVEGFVGAWIMTIIFAMILSNIMMRSKFFICPVN~D
AN5166.1  	NDIFAYVCGITFGRTQLIQLSPKKTVEGFLGAWVCTIIFGYFMTNILMRYKYFICPVY~D
          	***:**:*** *******:**********:***: *::*. :::::: * ::****.  *

MG09371.1 	LGANVFTGLSCDPNPAFLPTTWTMPDFVFLPRDFGKLSITMAPVQLHTLILSTFASLIAP
NCU09643.1	LGANIFTGLDCEPNPVFIPQTWTMPELLFFP-TKTRFSITMAPMQIHTFFWATFASLIAP
FG04237.1 	LGANIFTGLKCDPNPVFLPKTYEMPELFFLP-DTANFSFTIAPMQIHALNLATFASLIAP
AN5166.1  	LGSNVLTGLECDPNPVFVPQAFEIPEWTGFG-----RTFYVAPMQFHILLFATFASLVAP
          	**:*::***.*:***.*:* :: :*:   :       :: :**:*:* :  :*****:**

MG09371.1 	FGGFFASGLKRTFKIKDFGDSIPGHGGMTDRMDCQFTMGFFTYVYYHTFIATHKMGLGGI
NCU09643.1	FGGFFASGLKRTFKIKDFGHSIPGHGGITDRMDCQFIMGFFAYIYYHTFIAQSKVDFDEV
FG04237.1 	FGGFFASGLKRTFKIKDFGDSIPGHGGITDRMDCQFIMGFFAYMYFHTFVAIHKVSLGSV
AN5166.1  	FGGFFASGLKRSFKIKDFGESIPGHGGITDRMDCQFIMGFFAYMYYHSFIAVFKASVGDI
          	***********:*******.*******:******** ****:*:*:*:*:*  * ... :

MG09371.1 	MEDAITGLTPDEQIELIRGMGRYLANQGIVGAD0VLSCLDSALPMRR-
NCU09643.1	LEIVATRLTIDDHKKLLLFIAKYLVNHNAIDSS0IVNAIEKSLTNH--
FG04237.1 	LETAITSLNPEEQVELVKSMGHYLRNQGIVTED0SFSAKLYVYMLRVL
AN5166.1  	IEAAINGLTVDEQLEVLRGLSKYLYNQGAVPES0CLGDAYNL------
          	:* . . *. ::: :::  :.:** *:. :  .  ..
```
